# Effective partnership and in-country resource mobilization in Sudan for cVDPV2 outbreak response amid multiple emergencies in 2020–2021

**DOI:** 10.1186/s12889-023-15675-y

**Published:** 2024-01-19

**Authors:** Mohammed Taufiq Mashal, Dalya Eltayeb, Ariel Higgins-Steele, Ismael Suleiman El Sheikh, Ni’ma Saeed Abid, Hemant Shukla, Leonard Machado, Hamid Jafari

**Affiliations:** 1 Polio and Immunization Programmes, Sudan Country Office, World Health Organization, Khartoum, 2234 Sudan; 2 Federal Ministry of Health, Khartoum, 2234 Sudan; 3 Polio Eradication Department, Eastern Mediterranean Regional Office, World Health Organization, P.O. Box 811547, Mohammad Jamjoum Street, Ministry of Interior Building #5, Amman, 11181 Jordan; 4 Expanded Programme on Immunization (EPI), Federal Ministry of Health, Khartoum, 2234 Sudan; 5 World Health Organization, Sudan Country Office, Khartoum, 2234 Sudan

**Keywords:** Poliovirus eradication, cVDPV2 outbreak, Emergencies, Partnership, Domestic resource mobilization, Sudan

## Abstract

**Background:**

During 2020 and immediately prior to the COVID-19 pandemic, Sudan was experiencing multiple emergencies including violence, seasonal flooding, and vector-borne disease outbreaks. After more than ten years since its last case of wild poliovirus, Sudan declared a circulating vaccine-derived poliovirus type 2 (cVDPV2) outbreak on 9 August 2020.

**Methods:**

cVDPV2 outbreak response data and programme documents of the Federal Ministry of Health and WHO were reviewed. Surveillance data was verified through WHO-recommended procedures for detecting and characterizing polioviruses from stool and sewage samples collected from acute flaccid paralysis (AFP) cases and the environment.

**Results:**

This outbreak in Sudan led to a total of 58 confirmed cases of cVDPV2 from 15 of the 18 states. Two nationwide vaccination campaigns were held to increase immunity of children under-five against poliovirus type 2. Funding challenges were overcome by intense additional resource mobilization from in-country sources. The funding gap was bridged from domestic resources (49%) sourced through GPEI partners, and in-country humanitarian funding mechanisms.

**Conclusions:**

During an outbreak response and challenge of funding shortfall, mobilizing in-country resources is possible through coordinated approaches, regular communication with partners, disaggregation of needs, and matching in-kind and financial support to fill gaps. A cVDPV2 outbreak requires a fast, resourced, and quality response to stop virus circulation.

## Background

Countries and the Global Polio Eradication Initiative (GPEI) have made substantial progress toward polio eradication, though as of early 2020 – before the COVID-19 pandemic was declared – GPEI was not on-track to stop and prevent transmission of all three types of polioviruses by the target year of 2023 [1]. Two of the three wild polioviruses (types 2 and 3) have been eradicated, certified in 2015 and 2019, with type 1 remaining endemic in two countries [2].

After an extensive multi-year planning process, GPEI globally coordinated the cessation of all routine use of type 2-containing OPV (OPV2) in April–May 2016 [3]. OPV2 cessation represented an essential step toward the promise of a world free of type 2 polio, but unfortunately, OPV2 cessation did not end type 2 vaccine derived polio.

After OPV2 cessation, some countries reintroduced monovalent OPV2 (mOPV2) in response to outbreaks or environmental evidence of type 2 transmission [4]. In 2020, more than half of the 1078 global reported circulating vaccine-derived poliovirus type 2 (cVDPV2) cases occurred in 20 countries in Africa, which far exceeded the 140 reported global cases of type 1 wild poliovirus in 2020 [5, 6].

Although extremely rare, OPV can cause vaccine-associated paralytic polio in immunologically naive recipients or close contacts upon first exposure [7]. In addition, when used in populations with low immunization coverage, OPV can continue to circulate, instead of dying out, and lose its attenuating mutations as it spreads. Continued transmission of OPV-related viruses can lead to polio outbreaks caused by circulating vaccine-derived polioviruses (cVDPVs) that behave like wild-type polioviruses [7].

cVDPVs are not related to, nor indicative of a re-emergence of wild poliovirus. If a population is seriously under-immunized, there are enough susceptible children for the excreted vaccine-derived polioviruses to begin circulating in the community. Declining mucosal immunity following the global withdrawal of type 2 vaccine can require large and fast response to a cVDPV outbreak using a type 2 containing vaccine [8]. If a population is fully immunized against polio, it will be protected against the spread of both wild and vaccine strains of poliovirus [9].

Sudan initiated polio eradication activities in 1994 and reported its last indigenous wild poliovirus case in 2001. Interrupting periods of being polio-free, Sudan has experienced several importations of poliovirus; the last case of wild poliovirus in Sudan was detected in 2009 (type 1) [10–12]. Sudan has an established poliovirus surveillance system and network in all 18 states in the country which has been meeting globally established performance indicators [13].

After more than ten years since its last case of poliovirus, Sudan declared a cVDPV2 outbreak on 9 August 2020; the outbreak was confirmed as an importation associated with the CHD-NDJ-1 virus in neighboring Chad. The outbreak caused a total of 58 confirmed cases of cVDPV2 from 15 of the 18 states. Sudan’s Federal Ministry of Health (FMOH) followed the GPEI’s standard operating procedure to respond to the outbreak [14]. The FMOH and partners conducted a risk assessment which recommended the implementation of two nationwide house-to-house campaign rounds using mOPV2 targeting 8.45 million children under five years of age.

Our objective is to elaborate on how the Incident Management Team’s partnership – comprised of the World Health Organization (WHO) and United Nations Children’s Fund (UNICEF) – with Sudan’s Federal Ministry of Health, national and international partners succeeded mobilizing resources within the country for an effective cVDPV2 outbreak response.

## Methods

### Setting

Sudan is a signatory to the 1988 World Health Assembly resolution to eradicate poliomyelitis by the year 2000. The eradication efforts in Sudan started in 1994 following a wild virus outbreak in 1993.

The last Sudan endemic virus was reported from Unity State, southern Sudan in April 2001. This was followed by four wild poliovirus importations. The first importation was a wild poliovirus type 1 (WPV1) detected in Forbrunga in West Darfur State. It caused a total of 154 cases in 2004 and 2005 [15].

Then three importations occurred without causing any secondary case among the endogenous population: the second importation was one case of WPV1 in South Darfur State in 2007. The third importation was two cases WPV3 from West Darfur State in 2008. The fourth importation occurred 4 February 2009 due to WPV1 imported from the Republic of South Sudan.

The last case of wild polioviruses in Sudan was reported on 15 March 2009 from Red Sea state. The country remained free of any poliovirus case until 2020.

### Data collection and analysis

cVDPV2 outbreak response data and programme documents of the Federal Ministry of Health and WHO were reviewed. Surveillance data was verified through WHO-recommended procedures for detecting and characterizing polioviruses from stool and sewage samples collected from acute flaccid paralysis (AFP) cases and the environment. The poliovirus laboratory in Khartoum tests stool samples from AFP cases and environmental surveillance samples. Poliovirus positive samples are sent to a global polio lab network reference lab for genetic sequencing. All polio and non-polio samples are coded and tracked by unique numbers. Programme funding data available and gaps were tracked over time and in relation to the value of the Sudanese Pound against the United States Dollar (USD).

## Results

### Detection of cVDPV2 during COVID-19 pandemic

During the year 2020, Sudan faced several emergencies: episodes of intercommunal violence affected several parts of the country and especially in Darfur states, seasonal floods impacted all of the 18 states with over 900,000 people affected, vector-borne disease outbreaks such as malaria, dengue, and chikungunya occur annually in addition to the emergence of vaccine preventable diseases such as measles [16].

As of 9 February 2022, Sudan had reported 60,869 individuals confirmed positive for COVID-19, and 4,823 deaths to WHO [17]. As elsewhere in the region, the poliovirus eradication program engaged significantly in the COVID-19 response, including in the areas of rapid response and disease surveillance.

In mid-March 2020, the polio laboratory in Khartoum was repurposed for COVID-19 testing, which led to a delay in detecting the polio outbreak. During this period, while AFP cases continued to be reported, the collected stool samples were effectively stored in adequate conditions at a state level.

When the WHO Sudan team was notified of a cVDPV2 outbreak near the Sudanese border in Chad in June 2020, surveillance for AFP was immediately enhanced. Action taken included heightened sensitization of reporting sites, especially at border areas, and active case searches where possible. Country-led cross-border coordination between Sudan and Chad was enhanced and weekly calls between the two teams, in coordination with representatives from the WHO Eastern Mediterranean and African regions, and WHO Headquarters was established. Contacts of the key personnel in border areas were exchanged to ensure efficient cross-border collaborative work.

In the weeks after the global COVID-19 pandemic was declared in-country travel restrictions affected transport and the laboratory priorities shifted. As soon as the polio laboratory was reassigned for poliovirus testing on 22 July 2020, over 190 samples were moved to the lab within one week. Prioritization of testing was implemented to assess the samples, focusing on samples of cases of epidemiological interest. The first results were released by 7 August 2020.

COVID-19-related international travel restrictions also initially affected the transportation of AFP stool samples out of Sudan for advanced testing. Subsequently, samples were swiftly shipped to international reference laboratories for advanced testing and followed up for results.

### Outbreak epidemiology

The cVDPV2 detected in Sudan was genetically linked to an outbreak in neighboring Chad, indicating cross-border spread. The two countries worked together to share epidemiological information, surveillance and response strategies.

**Fig. 1:**
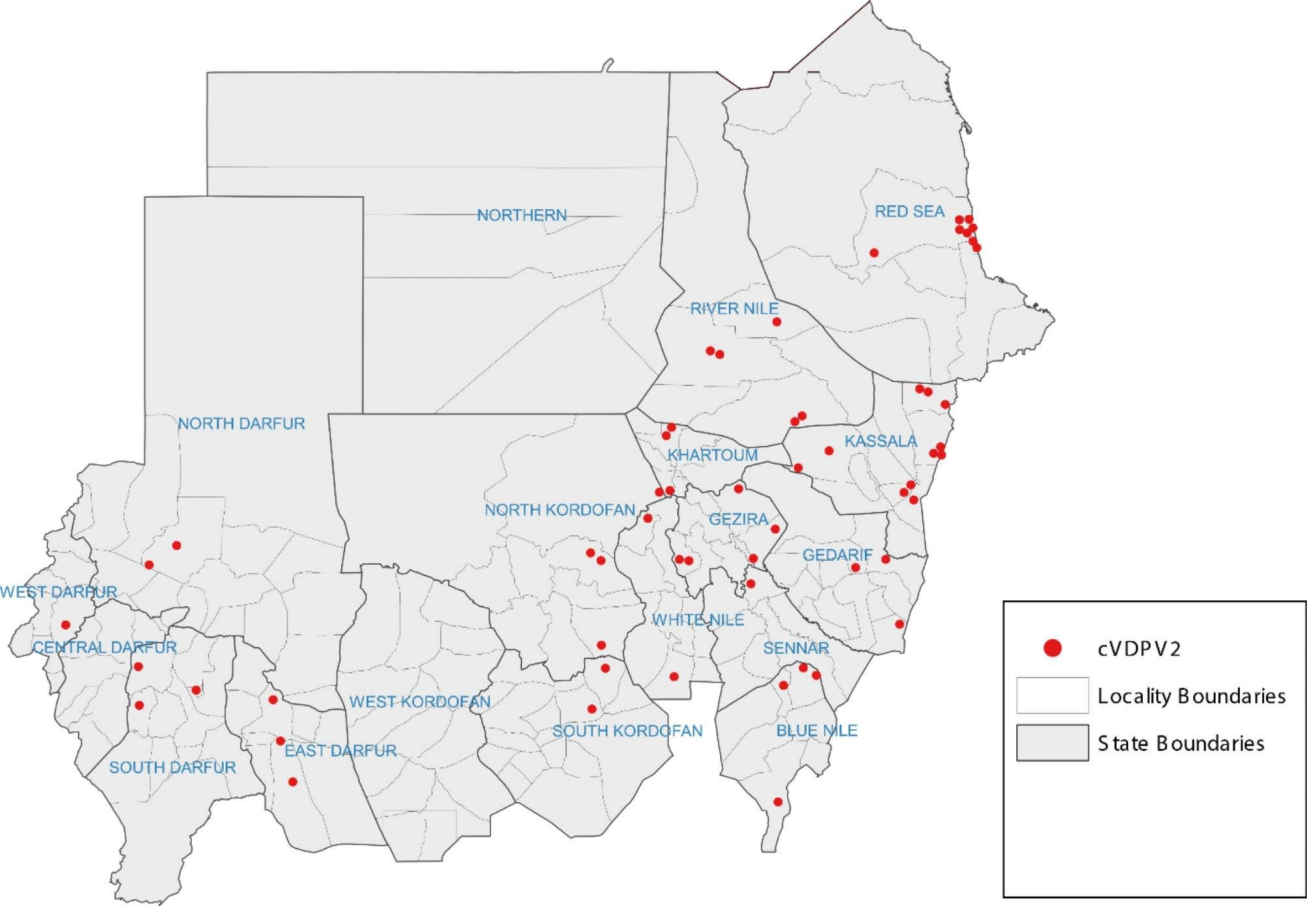
Distribution of cVDPV2 cases by states, Sudan 2020

**Fig. 2:**
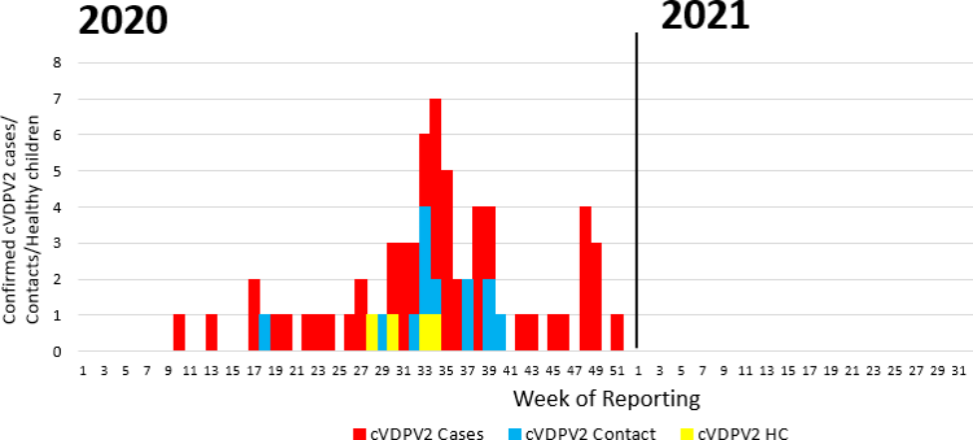
Epidemic curve of cVDPV2 cases, positive contacts, healthy children by week of reporting, Sudan

Sudan’s FMOH declared the outbreak of cVDPV2 on 9 August 2020 [18]. Over the course of the outbreak, Sudan reported a total of 58 confirmed cases from 15 out of 18 states (Fig. 1). The last confirmed case of this genetic lineage of cVDPV2 was detected in Sudan on 18 December 2020 (Fig. 2).

### Partnership and coordination

Following cVDPV2 outbreak notification, under the overall leadership of the FMOH, the WHO Sudan team with UNICEF established a GPEI Incident Management Team (IMT) to plan, coordinate, implement, and monitor overall cVDPV2 outbreak response management. The IMT followed the structure and approach that the WHO uses to manage response to public health events and emergencies [19]. The IMT for Sudan’s cVDPV2 response was co-led by the WHO and UNICEF offices in Sudan and included staff whose functional roles related to surveillance, vaccine-preventable disease and poliovirus eradication, vaccine management, communications, and social behavior change.

The IMT had weekly virtual calls and presented challenges and ways forward on outbreak response. With a risk assessment recommending the implementation of two nationwide house-to-house campaign rounds, the critical challenge was overcoming a budget deficit of USD 7.56 million to implement this outbreak response plan. The majority of the cost of the vaccination campaign (57%) was associated with transportation; fuel prices and incentives for campaign volunteers which had increased multi-fold due to the loss of the Sudanese pound value against the USD.

**Fig. 3:**
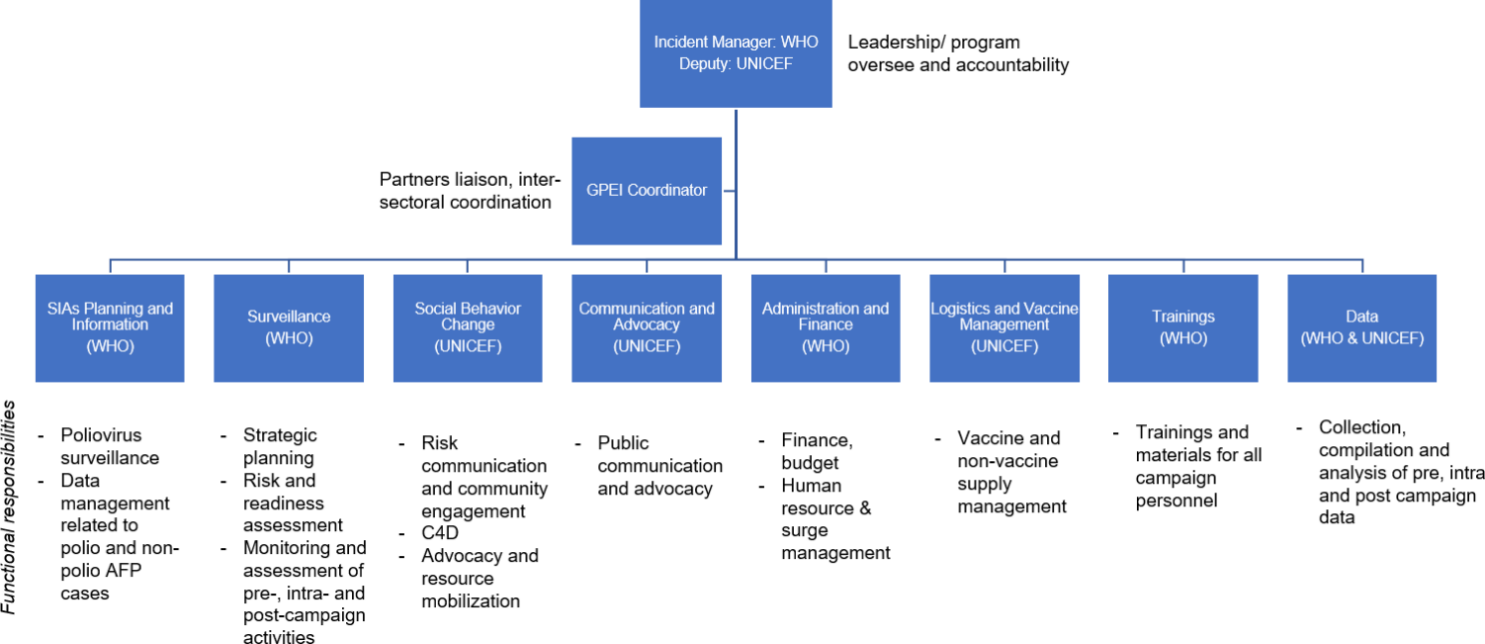
Structure of GPEI cVDPV2 Incident Management Team (IMT) in Sudan

The FMOH established an outbreak response steering committee and national technical committee. Several sub-national committees reported to the national technical committee, and a similar structure was functional at the sub-national levels (Fig. 3). The IMT was actively engaged with established structures at the FMOH and global GPEI. IMT members engaged United Nations Country Team (UNCT) and, through the UNCT, all health partners in Sudan. An active dialogue was opened between IMT, national, and international NGOs to share information on the polio outbreak given the absence of polio detections for more than a decade. In addition, the Health Cluster Coordination team received regular updates on the weekly call, including challenges and proposed solutions. The IMT used these coordination mechanisms to explain the cVDPV2 epidemiological developments and the plan for quickly increasing immunity for type 2 poliovirus through supplementary immunization activities (SIAs). As a core member of the IMT, WHO worked effectively with health partners through the Health Cluster Coordination forum to mobilize in-kind support to transportation by requesting vehicles, drivers, and fuel during the operation of vaccination campaigns.

As the primary outcome of these efforts, the United Nations Office for the Coordination of Humanitarian Affairs (OCHA) pledged to cover part of the financial gap. International NGOs promised provision of 98 vehicles, with drivers and fuel for five days implementation of vaccination campaigns; governors at the state level covered the gap of fuel and also provided food for mobile vaccination teams.

In coordination with GPEI, the IMT developed a clear roadmap of financial resources requirements for budget mobilization. GPEI funding totalled 13,449,369 USD, of which 11,705,789 USD was allocated for two national immunization campaigns. It was anticipated that none of the partners or government could single handedly cover the USD 7.56 million budget deficit. Therefore, the primary strategy was disaggregating the need and looking to diversify support.

The WHO polio-supported Public Health Officers followed the same partnership pattern in 14 out of 18 states. They coordinated the implementation of the outbreak response plan, provided technical support, and engaged state-level health partners.

### Economic situation and outbreak response funding

Loss of value of Sudanese pound against the USD and high inflation rate coupled with political instability and multiple outbreaks in the country were enormous challenges for the timely response of cVDPV2 outbreak in Sudan.

The WHO and UNICEF worked together with the FMOH and partners to support the government response to the outbreak to ensure every child under five years of age was vaccinated with the oral polio vaccine to improve immunity and protect against further spread of cVDPV2. Effective coordination and regular communication among the partners increased understanding of the outbreak’s impact and funding needs thus adding value of their financial contribution to the outbreak response.

Two rounds of vaccination campaigns with human resource surge support were planned with a total cost of USD 21,657,493 to offer children the best protection against type 2 circulating virus. The country program received USD 13,805,379 from the donors – based on the cost of the mass vaccination campaign in the country, national immunization days held in 2018 – which created a funding gap of USD 7,564,373. A major reason for this gap was difference in the UN exchange rate with open market and the high cost of fuel for transportation to implement two rounds of polio vaccination outbreak response campaign.

The IMT used multiple strategies to overcome the financial gap and ultimately fund the response. The IMT presented the case to in-country partners on several occasions, reviewed and revised activities in the outbreak response plan, repurposed the annual plan of polio eradication initiative activities, and called on the government of Sudan to translate their political commitment into financial commitment.

Through support from the FMOH and the Ministry of Finance (MOF), the Government of Sudan covered substantial financial gaps. WHO Sudan country office reprogrammed planned activities and budget to the operation cost of the new emergency of a cVDPV2 outbreak response.

**Fig. 4:**
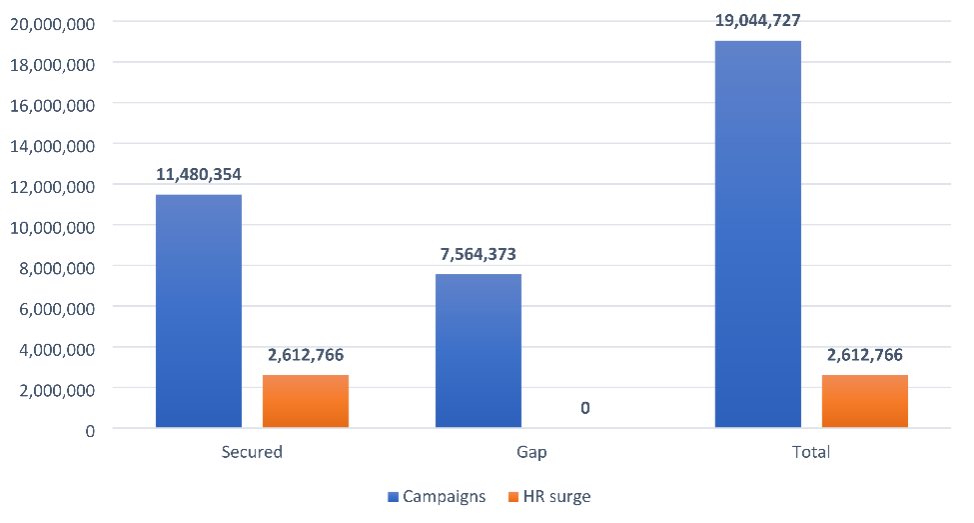
Total secured vs. gap (in US dollars) for implementation of two rounds of cVDPV2 outbreak response campaigns in Sudan, November 2020 and January 2021

As a result of coordination and systematic communication, the IMT overcame a USD 7,564,373 financial gap (Fig. 4). Several rounds of discussion with OCHA resulted in securing USD 1,506,000 from the Sudan Humanitarian Fund and moving USD 543,000 from the outbreak human resource surge plan to campaign operation cost. The GPEI partners’ Outbreak Preparedness and Response Task Team (OPRTT) contributed an additional USD 1,200,000. The IMT also reprogrammed support from Rotary International for a small-scale bivalent oral poliovirus vaccine (bOPV) SIAs to cover USD 631,000.

**Fig. 5:**
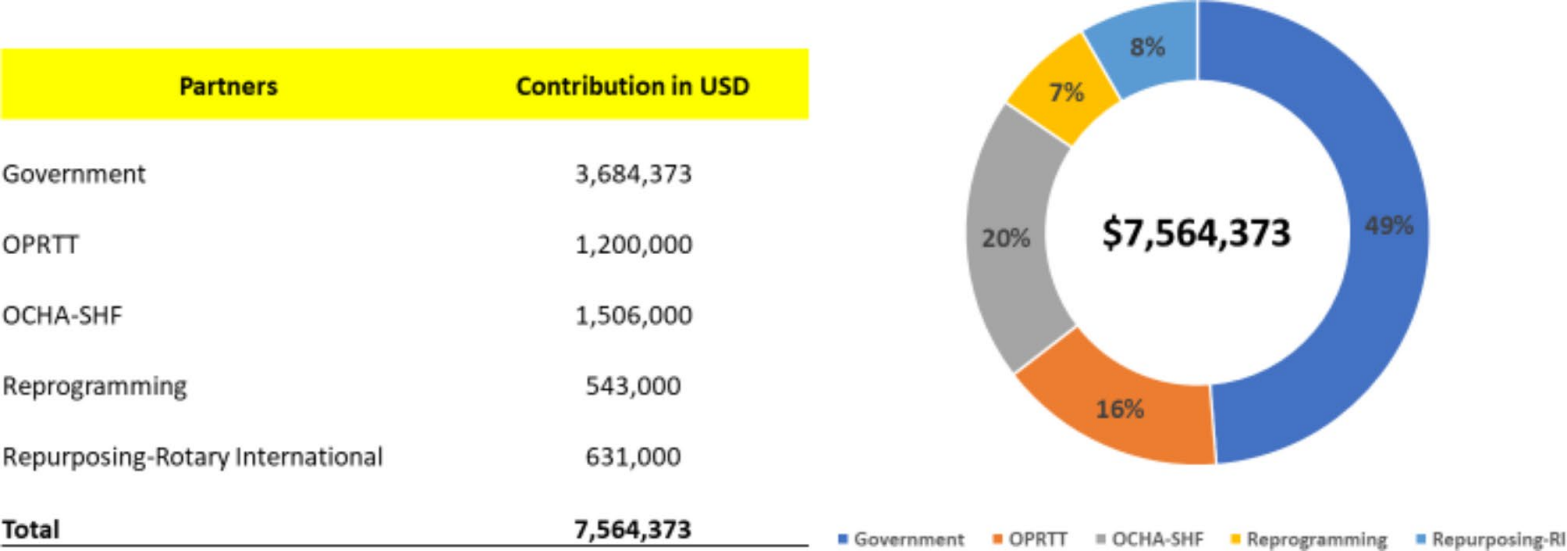
Partners’ financial contribution and percentage to support budget gap for implementation of cVDPV2 outbreak response campaigns in Sudan November 2020 and January 2021

Alongside GPEI donor funding, the government of Sudan was the main funder of the vaccination campaigns by domestic contributions of USD 3,684,373 which had not previously been earmarked for health, immunization, or emergencies (Fig. 5).

### Outbreak response

In the planning phase special attention was given to vaccination of high-risk and vulnerable population groups and areas. For the first time in ten years, immunization activities went ahead in Gorlangbang, in South Jebel Marra, a mountainous area in the southwest of the country which has been frequently affected by conflict, as well as in Ulu of Baw locality in Blue Nile state.

The country conducted two rounds of SIAs for the cVDPV2 using mOPV2 for children 0–5 years of age. The first round was conducted on 28 November 2020 reaching 97% (8.2 million) of 8.5 million children under five years of age targeted, according to the administrative coverage report. The second round was conducted in 17 states on 25 January 2021. The last state West Darfur conducted the second round on 22 February 2021.

**Fig. 6:**
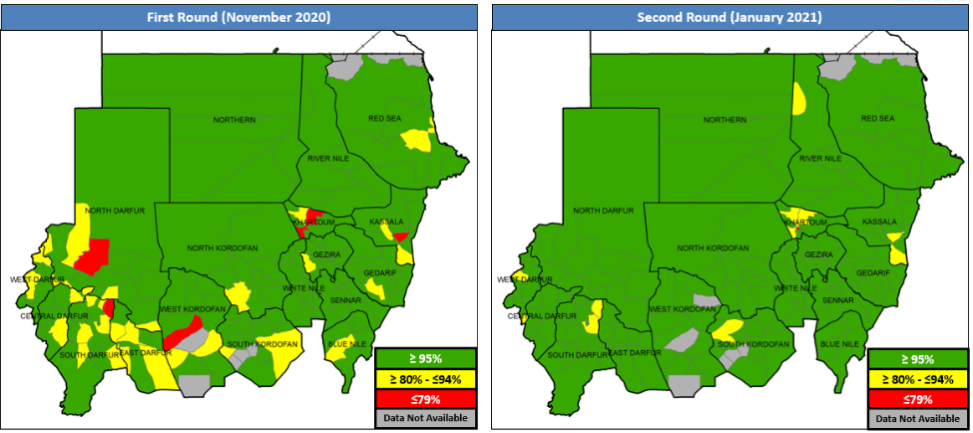
**SIA coverage by locality. **(source: administrative data)

The second campaign round reached 100% of target, 8.5 million children under five year of age or 98% according to independent monitoring survey drawn from 69,279 households. According to administrative data, fewer localities were below 95% coverage in second round and none were below 79% (Fig. 6). Targeting all children under five years with two doses of type 2-containing vaccine sought to quickly boost the immunity of children to protect against the cVDPV2. In addition, co-administration of Vitamin A took place in the second round, targeting 7.6 million children and achieving 99%.

In every location, vaccinators took infection prevention and control (IPC) measures against COVID-19, including washing hands and wearing masks.

### Surveillance

Sudan’s non-polio AFP rate at the state level has been on average above 3 per 100 000 children less than 15 years of age, and stool adequacy above 90% for 2019 and 2020, meeting global performance thresholds for these indicators. While the early phase of COVID-19 pandemic negatively impacted some surveillance indicators in the first half of 2020, the ability of the surveillance system to detect the outbreak shows that it remained sensitive. By end 2020, Sudan had identified a total of 733 AFP cases, which was higher than earlier years (608 in 2019, and 579 in 2018).

## Discussion

Our objective is to elaborate on how the Incident Management Team partnership with national and international partners in Sudan succeeded in mobilizing in-country resources for a nationwide cVDPV2 outbreak response.

The COVID-19 pandemic affected AFP surveillance and polio sample testing which resulted in delayed detection of the outbreak that spread to many states by the time the cVDPV2 outbreak was confirmed. COVID-19 also hampered routine immunization service delivery, and this exacerbated the immunity gap in vulnerable populations. Many children in Sudan are vulnerable to type 2 poliovirus. The primary strategy of the outbreak response was to raise immunity levels quickly, both through outbreak response activities and through encouraging uptake of routine and essential immunization services [8].

Sudan has a well-functioning surveillance system for communicable diseases such as polio. The AFP system proved to work well as it detected poliovirus circulation in both AFP cases and the environment. Surveillance indicators met global performance thresholds for non-polio-AFP rate and stool adequacy during this time period.

Following detection of the outbreak, surveillance for all kinds of poliovirus was enhanced with the aim of ensuring a non-polio AFP detection rate of 3 and above per 100,000 children less than 15 years at state and locality (i.e. district) levels. Despite challenges caused by COVID-19 restrictions, indicators remain above global standards. In most states, Sudan is meeting both key indicators most commonly used to assess the polio surveillance system: the non-polio AFP rate and stool adequacy.

The IMT developed an outbreak response plan and identified the financial need for vaccination campaigns and human resources surge support. The plans for the vaccination campaigns provided details of the cost for human resources, transportation, vaccine management, communication, social mobilization, and post-campaign monitoring. Breakdown of response components allowed partners to focus on their area of interest for funding contribution.

Polio programme management used multiple strategies to overcome the financial gap. This was presented to the in-country partners on several occasions, which resulted in reprogramming activities in the outbreak response plan, replanning annual polio eradication initiative activities, and calling on the government of Sudan to translate their political commitment into action.

Donors fully funded the surge but there was a gap in the operation cost of the two vaccination campaigns. The implementation of various innovative strategies to increase locally mobilized funds increased the cumulative funding requirements under the IMT responsibility from USD 13,805,379 to USD 21,657,493. Advocacy for resource mobilization will remain critical for polio eradication particularly when response costs are underestimated [20].

The transparent use of resources and systematic implementation of the planned activities in a low-resource context with multiple emergencies reinforces confidence among national counterparts and donors. With new emphasis on non-polio specific outcomes and integration alongside the core polio eradication agenda, improved reporting related to costs and programme performance is paramount [21].

Government and polio eradication initiative partners should maintain and even strengthen the local resource mobilization machinery, widen its donor base, and step up advocacy to ensure that Sudan successfully continues its polio-free status. Flexibility in approvals for utilization of funds which was demonstrated with the reprogramming of Rotary funds earmarked for catch-up campaigns for the outbreak response contributes immensely to such circumstances.

Proactively and regularly engaging government stakeholders and third-party influencers to ensure consistent understanding of the challenges and actions is needed to end cVDPV2 outbreaks. It is important to listen and collect feedback on how the GPEI can better support governments, and to promote joint accountability in the GPEI’s partnerships with governments to end all forms of polio [22].

As part of polio transition planning, we also recommend that the local resource mobilization mechanism be applied to support routine and essential immunization, introduce new vaccines, and strengthen health systems in Sudan [23]. In further alignment with the Immunization Agenda 2030 (IA2030) and Gavi, the Vaccine Alliance’s strategic plan 2021–2025 (“Gavi 5.0”), the new strategy offers a more holistic approach to immunization and shares with IA2030 its principles of being people-centered, country-owned, partnership-based and data-guided [24].

Although domestic resource mobilization is common, this experience demonstrated interventions to mobilize resources for a public health emergency in a country with multiple emergencies and severe economic crisis is possible. A coordinated, holistic approach and systematic communication with all stakeholders in the country facilitates better resource mobilization, and underpinned a fast and effective response to a new cVDPV2 outbreak.

## Conclusion

During an outbreak response and challenge of funding shortfall, mobilizing domestic resources is possible through coordinated approaches and systematic communication with in-country partners, disaggregation of needs, and diverse interventions. A cVDPV2 outbreak requires a fast, resourced, and quality response to stop virus circulation.

## Data Availability

The datasets generated during and/or analyzed during the current study are available from the corresponding author on reasonable request.

## Abbreviations

AFP: acute flaccid paralysis
bOPV: bivalent oral poliovirus vaccine
cVDPV2: circulating vaccine-derived poliovirus type 2
FMOH: Sudan’s Federal Ministry of Health
GPEI: Global Polio Eradication Initiative
IA2030: Immunization Agenda 2030
IMT: Incident Management Team
MOF: Sudan’s Ministry of Finance
mOPV2: monovalent oral poliovirus vaccine
OCHA: United Nations Office for the Coordination of Humanitarian Affairs
OPV: oral poliovirus vaccine
OPV2: oral poliovirus type 2 containing vaccine
OPRTT: GPEI’s Global Outbreak Preparedness and Response Task Team
SIAs: supplementary immunization activities
UNCT: United Nations Country Team
UNICEF: United Nations Children’s Fund
USD: United States Dollar
VDPVs: vaccine derived polioviruses
WHO: World Health Organization
WPV1: wild poliovirus type 1
